# Changes in Stress, Coping Styles, and Life Satisfaction between the First and Second Waves of the COVID-19 Pandemic: A Longitudinal Cross-Lagged Study in a Sample of University Students

**DOI:** 10.3390/jcm10174025

**Published:** 2021-09-06

**Authors:** Aleksandra Maria Rogowska, Cezary Kuśnierz, Dominika Ochnik

**Affiliations:** 1 Institute of Psychology, Faculty of Social Sciences, University of Opole, 45-052 Opole, Poland; 2 Faculty of Physical Education and Physiotherapy, Opole University of Technology, 45-758 Opole, Poland; c.kusnierz@po.edu.pl; 3 Faculty of Medicine, University of Technology, 40-555 Katowice, Poland; dominika.ochnik@wst.pl

**Keywords:** avoidance-oriented coping, college students, coping styles, emotion-oriented coping, life satisfaction, perceived stress, task-oriented coping, satisfaction with life, university students

## Abstract

In this study, we aimed to explain the interplay mechanism between stress, life satisfaction, and coping styles among university students. A cohort study was performed during the first (wave 1; W1) and second (wave 2; W2) waves of the Coronavirus disease 2019 (COVID-19) pandemic. The total sample included 231 university students, of which 59.31% were women. The Satisfaction with Life Scale (SWLS), Perceived Stress Scale (PSS), and Coping Inventory for Stressful Situations (CISS) were included in one online survey. Stress, emotion-oriented, and avoidance-oriented coping styles increased from W1 to W2 of the COVID-19 pandemic, while life satisfaction and task-oriented coping decreased. The partial mediation effect of all three coping styles during W1 and W2 (in a cross-sectional approach) on the relationship between perceived stress and life satisfaction was confirmed in this study. The task-oriented and emotion-oriented coping styles can play a mediating role in the reciprocal relationship between life satisfaction and perceived stress during W1 and W2 of the pandemic. There were no mutual interactions between stress and life satisfaction from a longitudinal approach. Coping styles changed subsequently due to stressful environmental changes related to lockdown during the COVID-19 pandemic. Having a wide range of coping strategies from which to choose during an unstable situation should help manage stress and well-being.

## 1. Introduction

Satisfaction with life can be defined as the global cognitive self-judgment of well-being across a broad set of human activities at school, work, with family, and in social life [1]. Life satisfaction is considered a significant predictor of mental and physical health and successful adaptation to life [2,3,4,5]. Research indicates that higher levels of perceived stress are related to decreased levels of life satisfaction [4,6,7,8,9,10,11,12,13,14]. Numerous studies reported a decrease in well-being and increases in distress, loneliness, insomnia, anxiety, and depression during the coronavirus outbreak [15,16,17,18,19,20,21,22,23,24,25,26,27,28,29,30,31,32,33,34,35,36]. Physical and mental health was found to be a significant positive predictor of life satisfaction during the pandemic [37]. The most desirable skill in a pandemic situation seems to be coping strategies aimed at regulating and reducing negative emotions or pessimistic and unrealistic thinking, as well as solving current problems related to quarantine restrictions, isolation, job loss, deterioration of economic status, and countless lifestyle changes.

In Poland, lockdown started 10–12 March 2020 (with the closing of schools and universities), expanded on 25 March (to limiting non-family gatherings to two people and forbidding non-essential travel), and restrictions tightened on 31 March. Starting from 30 May 2020, wearing masks in outdoor places was no longer obligatory but was restored as of 10 October 2020 due to the increasing number of cases. Primary lockdown-related stress sources were as follows: isolation, restriction in moving, shopping, traveling, changes in daily lifestyle regards use of face masks, washing hands frequently, avoiding social contact and gatherings, restaurants, pubs, clubs, fitness clubs, limiting physical activity outdoors, and required remote online learning and work [17,38,39,40,41,42,43].

One of the largest sources of stress was an economic crisis due to prolonged lockdown, which increased concerns about future work-finding and financial stability [18,44]. Lee [45] showed that perceived employment and housing insecurity, deteriorating finances, and difficulties in paying for basic needs predicted life satisfaction, happiness, health self-esteem, mental health index, and mental stress among a large sample of European Union citizens. Remote online learning and academic stress were the risk factors for mental health and decreased well-being of university students before quarantine [46,47] as well as during the pandemic [48,49,50,51]. In the present study, university students were examined regarding perceived stress, coping styles, and life satisfaction during the Coronavirus disease 2019 (COVID-19) pandemic.

### 1.1. Association between Stress, Coping, and Subjective Well-Being

Coping strategies seem to play a pivotal role in physical and mental health, particularly during adaptation to stressful situations in life [52]. According to the transactional theory of stress [53], stress is understood as a relationship between an individual and their environment, which the person appraises as relevant to their well-being. Stress can emerge when an individual perceives an exceeding of their resources to cope. Lazarus [54] (p. 99) defined coping with stress as “constantly changing cognitive and behavioral efforts to manage specific external and/or internal demands that are appraised as taxing or exceeding the resources of the person”. Coping has two main functions: regulating disturbing emotions (aimed at regulating emotional distress) and focusing cognition and behavior on solving the problem that causes distress (aimed at altering person–environment relationships).

Recent international research [55] performed in Russia, Kyrgyzstan, and Peru during the COVID-19 pandemic found that the coping responses related to problem-focused coping, socially supported coping, avoidance, and emotion-oriented coping explained 44% of the coping variability. Significant differences in religious coping and mental disengagement were found across the countries, suggesting that some coping behaviors may play distinct roles in responding to stressful events. Higher psychological distress was associated with more frequent use of passive (negative) coping, but less frequent use of an active (upbeat) coping style during the COVID-19 pandemic [56,57]. There was also an association between coping with stress and subjective well-being. In particular, task-oriented coping style was related positively to well-being, whereas emotion-oriented and avoidance-oriented coping strategies were related negatively [35,58,59,60,61,62,63,64].

### 1.2. The Theoretical Background of the Current Study

Studies from various regions of the world indicate that about 50% of people have experienced high stress levels during the coronavirus pandemic [65,66,67,68]. Khodami [24] found that younger people and individuals with a low quality of life were more likely to experience higher stress levels and more significant emotion regulation problems. With increasing quarantine time, quality of life decreased, and perceived stress and emotion regulation problems increased. From a biocultural perspective, financial crisis and prolonged emotional stress during the COVID-19 pandemic may substantially impact growth and development for the next generation, as suggested by Bogin and Varea [69]. Therefore, research on the factors that may decrease stress and elevate well-being in young adults is currently necessary to prepare adequate support, intervention strategies, and prevention programs.

In this study, we examined life satisfaction, stress, and coping style during the coronavirus-related lockdown, considering an intra-individual approach to coping across two stressful situations: the first and second waves of the COVID-19 pandemic. Previous research indicated that university students experience low life satisfaction, high levels of perceived stress, and emotion-oriented coping styles during the first wave of the COVID-19 pandemic [30,70,71]. Life events and coping are intertwined, as reported from studies on the mediating role of coping in the relationship between stress and imminent health consequences [72].

According to the cognitive-transactional model of stress [73], stress results from an interaction between a person and their environment and is subject to continuous change. The meaning of a particular stressful person–environment transaction is derived from the underlying context. Both coping and cognitive appraisals of demands and resources can play a mediating role in the association between experiencing stress and psychological well-being. Coping includes emotional (affective) components that cause physiological changes and have long-term effects concerning mental and somatic health and well-being, and social functioning. The coping strategy is selected as a result of the appraisal process. Lazarus and Folkman [53] suggested that the primary appraisal determines whether a situation is stressful, and a secondary appraisal is initiated to assess the situation, select an appropriate coping strategy, assess the likelihood that a coping option will achieve the expected effect, and whether the person can effectively apply the strategy. In the transactional process, people can continuously reappraise the situation as coping strategies are initiated and the person–environment relationship changes. The short- and long-term health-related outcomes of the process are determined by the selected coping strategy and may vary depending on the setting.

Lardier et al. [74] found a mediating effect of reflective (task-oriented) coping on the relationship between perceived stress and life satisfaction in a cross-sectional study among Hispanic undergraduate students. Reverse mediation analysis, with life satisfaction as a predictor of stress, was also performed [75]. The transactional model of stress [53,73] assumes a one-way relationship from stress (predictor) to life satisfaction (outcome). However, if adverse changes in the outcome (i.e., life satisfaction) are perceived as a stressor, this may trigger a primary appraisal and restart the complete transactional process. Therefore, the inverse model investigated by Gori et al. [75] seems to be equally likely. Here, we examined the interrelationship between stress and life satisfaction and coping styles as mediators of these relationships during the COVID-19 pandemic. Based on the previous studies described above, we formulated the following hypotheses:

**Hypothesis** **1** **(H1):** *Changes occurred between the first (W1) and second (W2) waves of the COVID-19 pandemic in perceived stress, life satisfaction, and coping styles, as a consequence of stressful person–environment transactional process* [53,73].

**Hypothesis** **2** **(H2):** *Life satisfaction at W2 may be explained by perceived stress and coping style during W1 and W2 and by life satisfaction measured during W1*.

**Hypothesis** **3** **(H3):** *Coping styles play a mediating role in the relationship between stress (predictor) and life satisfaction (outcome) during the first (W1) and second (W2) cross-sectional measurements* [74].

**Hypothesis** **4** **(H4):** *A reciprocal relationship exists between life satisfaction as a predictor and stress as an outcome, and coping styles as mediators, during the first (W1) and second (W2) cross-sectional measurements* [75].

**Hypothesis** **5** **(H5):** *Because the theory of transactional stress process [53,73] assumes continuous changes in stress and coping, the coping styles during W1 cannot predict either perceived stress during W2 or life satisfaction during W2 using a longitudinal approach*.

## 2. Materials and Methods

### 2.1. Study Design

**Figure 1:**
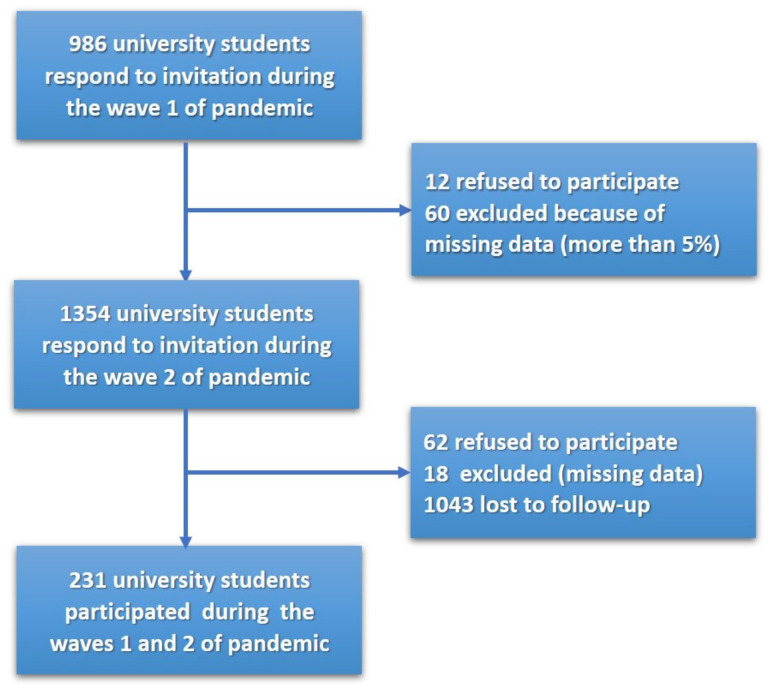
Flow chart of the study sample by recruitment strategy during each wave of the COVID-19 pandemic.

A cohort study was performed in two waves. The first wave of cross-sectional study was conducted in spring 2020 and the second wave in autumn 2020, at a large public technical university in the south of Poland. The necessary sample size was determined using G*Power ver. 3.1.9.4. Software [76,77]. The sample size equaled 167 people, if calculated a priori for bivariate correlation with *r* = 0.30, *p* < 0.01, and 95% CI, and equaled 108 people for a linear multiple regression model with two independent variables, effect size *f*^2^ = 0.15, *p* < 0.01, and 95% CI. University students were recruited through the online e-learning platform at a university. The invitation to participate in the study (with a link to the on-line survey) was provided on the Moodle platform from 3 March to 29 April 2020, during the first wave (W1) of the COVID-19 pandemic. The information about the study was provided and informed consent was obtained using the first page of the online questionnaire. Students were informed that participation was voluntary and they could refuse from the survey at any time. No form of compensation was offered as an incentive to participate. The average time for data collection was 20 min. The student sample was highly diverse to minimize sources of bias due to their key characteristics: field of study and study cycle. Among the university students, 986 people responded to the invitation during W1, but 12 refused to participate, and 60 presented more than 5% missing data, so they were excluded from further statistical analyses. Altogether, 914 students participated at measurement time one (W1) and completed all measures, including life satisfaction, perceived stress, and coping with stress (Figure 1).

The same procedure was used during the second wave (W2) of the COVID-19 pandemic. The research was conducted from 3 November to 3 December 2020. Among university students, 1354 responded to the invitation. However, 62 refused to participate during W2, and 18 were excluded because more than 5% of their data was missing. Among the 1274 university students during W2, 231 participants matched the following demographic characteristics from W1: birth date, sex, place of residence, faculty, level, and year of the study (Figure 1). Therefore, 231 university students were included in the total sample that was examined using all statistical tests.

The Research Ethics Committee approved the study protocol at the University of Opole, Poland (1/2020). The study followed the ethical requirements of anonymity and voluntariness of participation. Following the Declaration of Helsinki, written informed consent was obtained from each student before inclusion. We received no specific funding for this work. This study is part of an international research project, “Well-being of undergraduates during the COVID-19 pandemic: International study”, registered at the Center for Open Science (OSF) [78].

### 2.2. Participants

The participants in the study were 231 university students, aged between 21 and 37 years (M = 23.21, SD = 2.28), with a prevalence of women (*n* = 137, 59%), those living in villages (*n* = 107, 46%), studying physical education and physiotherapy (*n* = 72, 31%), in first level (Bachelor, *n* = 174, 75%), involved in full-time education (*n* = 200, 87%), and second-year of study (*n* = 102, 44%). More details about the demographic characteristics of the students during W2 of the COVID-19 pandemic are listed in Table 1.

### 2.3. Measurement

#### 2.3.1. Perceived Stress

The Perceived Stress Scale (PSS) was developed for measuring psychological stress [79]. This is a self-report ten-item questionnaire, with a 5-point Likert scale (ranging from 0 = never to 4 = very often). The participant indicates how often they experienced a given type of behavior in the last month. Total scores range between 0 and 40, with higher scores indicating higher levels of perceived stress. The scores ranging between 5 and 11 indicate extremely low stress, 12–17 indicates low, 18–23 average, 24–28 high, and 29–35 extremely high stress. The internal consistency of the PSS-10 assessed by Cronbach’s α was 0.88 during W1 and 0.90 during W2.2.3.2. Coping styles.

The Coping Inventory for Stressful Situations (CISS) was developed by Endler and Parker [80] on theoretical and empirical bases to provide a self-report measure of responses to stressful circumstances. The CISS consists of 48 items in three scales (16 items in each dimension): task-oriented, emotion-oriented, and avoidance-oriented coping styles. Task-oriented coping relies on restructuring and focusing on tasks, problem solving, altering the situation, and planning. An emotion-oriented coping style involves self-oriented emotional reactions in stressful situations (e.g., self-preoccupation, self-blaming, upset, getting angry, becoming tense, and fantasizing). Avoidance-oriented coping involves using distractions by other situations or tasks or social gatherings. Respondents rated on a 5-point Likert scale (1 = not at all to 5 = very much) the degree of engagement in various types of activity during a difficult, stressful, or upsetting situation. Higher scores are interpreted as greater use of the coping style. In the present study, the reliability (Cronbach’s α) for task-, emotion-, and avoidance-oriented coping was 0.91, 0.91, and 0.80 during W1, and 0.93, 0.92, and 0.86 during W2, respectively.

#### 2.3.2. Life Satisfaction

The Satisfaction With Life Scale (SWLS) is a short 5-item scale developed by Diener et al. [1] to assess global cognitive judgments of one’s life satisfaction. An individual chooses how much they agree with a given item on a seven-point Likert scale (1 = strongly disagree to 7 = strongly agree). Higher scores indicate a higher level of life satisfaction, ranging from 5–9 = extremely dissatisfied, 10–14 = dissatisfied, 15–19 = slightly dissatisfied, 20 = neutral, 21–25 = slightly satisfied, 26–30 = satisfied, and 31–35 = extremely satisfied. The Satisfaction with Life Scale (SWLS) provides evidence of a stable one-factor structure, high reliability, validity, and invariance for sex [1,11,12,81,82,83]. The Cronbach’s α for the SWLS in the present sample was 0.81 during W1 and 0.85 during W2.

### 2.4. Statistical Analysis

Missing data were handled by mean imputation, but if exceeding 5%, the case was removed from further statistical analysis. In the preliminary analysis, descriptive statistics were calculated for all measures, including the mean (M), 95% CI, standard deviation (SD), median, skewness, and kurtosis. All scores demonstrated good parametric properties. Therefore, further parametric analyses were conducted. The study hypotheses were tested in several ways. Repeated measures one-way ANOVA was used to examine changes in the mean scores of life satisfaction, perceived stress, and coping styles during the first and second waves of the COVID-19 pandemic. Effect sizes were calculated using the partial eta-squared statistic (η*_p_*^2^).

The Pearson’s correlation matrix was used to test the bivariate correlations among the variables. Multiple linear regression was conducted to assess the association between life satisfaction during W2 as a dependent variable and perceived stress and coping styles during W1 and W2 of the COVID-29 pandemic as a predictor variable. The mediating role of coping styles in the relationship between perceived stress and life satisfaction was examined in a cross-sectional design, separately for the first and second pandemic waves, using structural equation modeling (SEM). Parallel mediation models (simultaneous analysis of all three coping styles as a mediator) were performed based on maximum likelihood (ML) estimation without missing values and including observed variables. The conditional effect was examined based on a bias-corrected bootstrapping procedure with 1000 samples. A bootstrap confidence interval (95% CI) not including 0 signaled a significant effect.

Next, a two-wave cross-lagged panel design with a time lag of a half-year between W1 and W2 was performed for testing the prospective effect of perceived stress and coping styles on life satisfaction [84]. Two waves of data collection are recommended as the optimal approach to longitudinal design because it curtails the cost of a study in terms of time and money [85]. Structural equation modeling (SEM) was used, based on maximum likelihood (ML) estimation, without missing values, including observed variables, and with the bootstrapping method to test the conditional effect. The parallel mediation model specified included ten observed variables. We decided not to use latent variables because of the problems related to measurement invariance for the total 126 items included in 10 variables (W1 and W2). Both cross-lag and autoregressive paths were used to examine stability and change simultaneously [86,87]. All latent structural models were evaluated using several goodness-of-fit criteria, including ML χ^2^, *df,* and *p*-value (the ratio χ^2^/*df* < 5 representing a good fit), standardized root mean square residual (SRMR ≤ 0.08 indicates an acceptable fit), root mean square error of approximation (adequate fit if RMSEA ≤ 0.08), comparative fit index (CFI ≥ 0.90 meaning adequate fit), normed fit index (NFI ≥ 0.95 considering adequate fit), and the Tucker–Lewis index (TLI ≥ 0.90 indicating adequate fit) [88,89,90]. All statistical analyzes were conducted using SPSS 27 (for descriptive statistics, ANOVA, and correlation analysis) and AMOS 22 (for SEM).

## 3. Results

### 3.1. Differences between the First and Second Waves of the COVID-19 Pandemic

**Figure 2:**
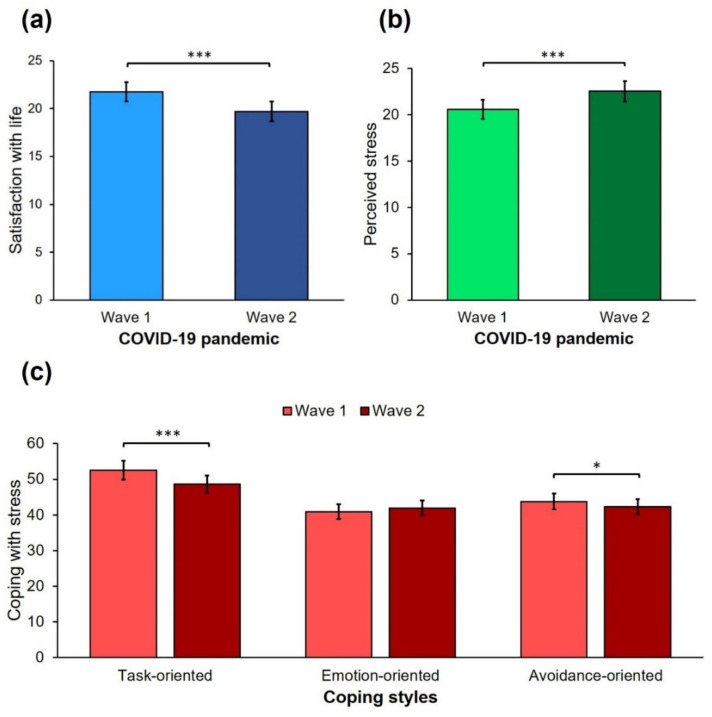
Differences between the first and second waves of the COVID-19 pandemic in (**a**) satisfaction with life; (**b**) perceived stress; and (**c**) task-oriented, emotion-oriented, and avoidance-oriented coping styles. * *p* < 0.05, *** *p* < 0.001.

The repeated measures one-way ANOVA was performed separately for life satisfaction, perceived stress, and coping styles to examine differences between the first (W1) and second (W2) wave of the COVID-19 pandemic among university students. The results are presented in Table 2 and Figure 2. The sample of university students reported an average (neutral) level of life satisfaction during the second wave of the pandemic (range 5–34, M = 19.70, SD = 6.11). Among participants, 11 individuals (4.76%) were extremely dissatisfied, 41 (17.75%) were dissatisfied, 58 (25.11%) were slightly dissatisfied, 14 (6.06%) were neutral, 68 (29.44%) were slightly satisfied, 30 (12.99%) were satisfied, and 9 (3.90%) were extremely satisfied. Perceived stress was reported as average (range 2–39, M = 22.53, SD = 7.92). In the sample, 45 persons (8.66%) indicated extremely low stress, 55 (19.48%) low, 53 (23.81%) average, 58 (22.94%) high, and 20 (25.11%) reported extremely high stress. The mean results of coping styles are presented in Table 2.

Significant differences with a medium effect size were found in life satisfaction and task-oriented coping style, as well as in perceived stress and avoidance coping with a small effect size. However, considering the Bonferroni correction, the level of significance was above the threshold of *p* < 0.01 for the avoidance coping style, which means that the differences between the first and second waves of pandemic should be considered statistically non-significant. No differences were found in the emotional coping style between W1 and W2.

### 3.2. Examining Predictors of Life Satisfaction at W2

In a preliminary analysis, Pearson’s correlation was calculated to examine the association between satisfaction with life, perceived stress, and coping styles during the first and second waves of the COVID-19 pandemic. Almost all variables were related to each other, as shown in Table 3. The regression analysis was performed for life satisfaction during the second wave of the coronavirus pandemic (as a dependent variable) and predictor variables such as life satisfaction, perceived stress, and coping styles during the first and second wave.

Among all variables included in the regression model, the significant predictors were life satisfaction and perceived stress during the first wave of pandemic and perceived stress, task-oriented, and emotion-oriented coping styles during the second wave (Table 4). The model of regression explained 70% of the life satisfaction variance (*R*^2^ = 0.70, *F*(9, 221) = 24.09, *p* < 0.001).

### 3.3. The Indirect Effect of Perceived Stress on Life Satisfaction via Coping Styles

**Figure 3:**
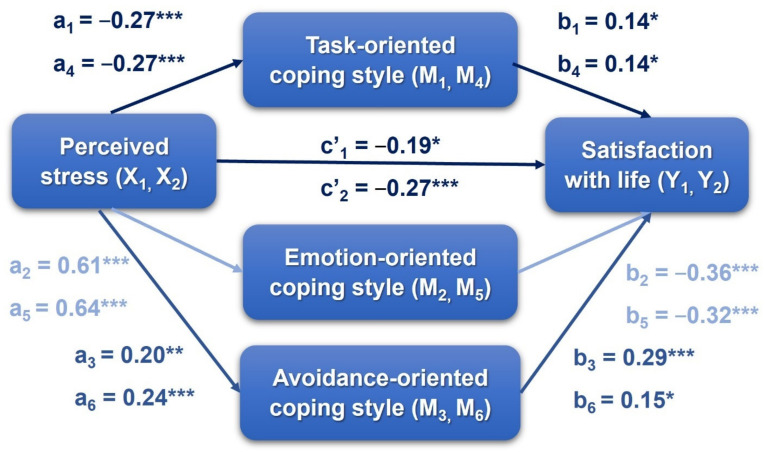
Path model of mediation of the effect of perceived stress on satisfaction with life, via coping styles; cross-sectional design (Model 1 and Model 2). * *p* < 0.05, ** *p* < 0.01, *** *p* < 0.001.

Cross-sectional parallel mediation analysis was conducted to examine the simultaneous mediation effect of all three coping styles on the relationship between perceived stress and life satisfaction (Figure 3). The analysis was performed separately for wave 1 (Model 1) and wave 2 (Model 2) of the COVID-19 pandemic. As shown in Table 5, all standardized estimates were statistically significant at both W1 and W2, which confirms the indirect effect of perceived stress on life satisfaction via task-oriented, emotion-oriented, and avoidance-oriented coping styles.

The goodness-of-fit indices for the cross-sectional parallel mediation during W1 (Model 1) and W2 (Model 2) are shown in Table 6. Some indices show good fit (i.e., SRMR, CFI, and NFI), whereas others are less acceptable (ML Χ^2^/*df*, RMSEA, and TLI).

### 3.4. The Indirect Effect of Life Satisfaction on Perceived Stress via Coping Styles

**Figure 4:**
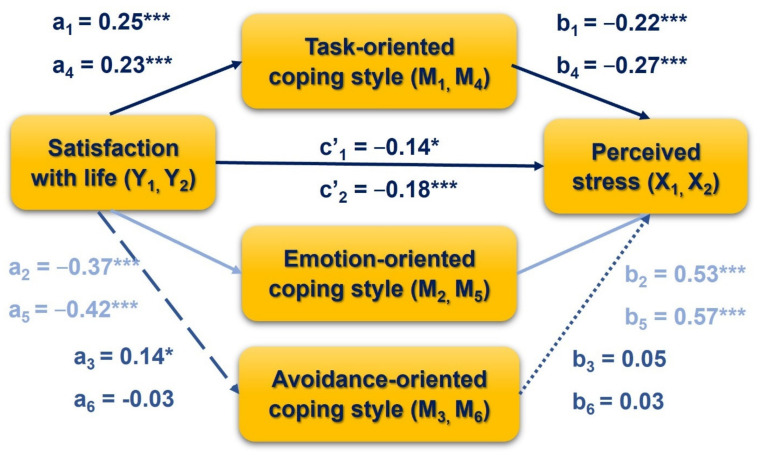
Path model of mediation for the effect of perceived stress on satisfaction with life, via coping styles; cross-sectional design (Model 3 and Model 4). * *p* < 0.05, *** *p* < 0.001.

The cross-sectional parallel mediation analysis was performed separately for W1 and W2 to test the indirect effect of life satisfaction on perceived stress via coping styles (Figure 4). As is shown in Table 7, task-oriented and emotion-oriented coping (but not avoidance-oriented) were found as mediators in the association between life satisfaction and perceived stress for both W1 and W2. Table 6 demonstrates the goodness-of-fit indices for Model 3 (W1) and Model 4 (W2), which are the same as Model 1 and Model 2. According to the goodness-of-fit criteria, Model 3 and 4 (similar to Model 1 and 2) presents a satisfactory fit, taking into account SRMR (<0.08) CFI (>0.96), NFI (>0.95), and less acceptable for ML Χ^2^/*df* (>5 is unacceptable), RMSEA (acceptable <0.08 in Model 1 and 3, but unacceptable >0.08 for Model 2 and 4), and TLI (<0.90 is unacceptable).

### 3.5. Longitudinal Mediating Role of Coping Styles Using a Cross-Lagged Panel Model

**Figure 5:**
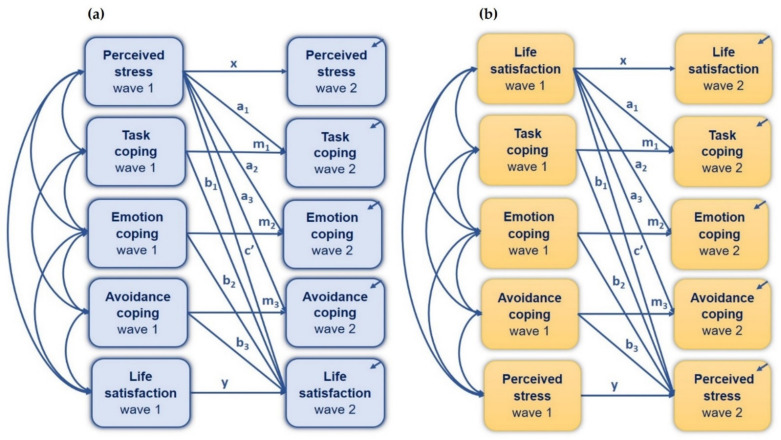
Conceptual cross-lagged path model for a prospective study on the parallel mediation role of coping styles in the relationship between perceived stress and satisfaction with life during the first and second waves of the COVID-19 pandemic: (**a**) perceived stress as a predictor of life satisfaction (Model 5); (**b**) life satisfaction as a predictor of perceived stress (Model 6).

The indirect effect of perceived stress on life satisfaction via coping styles was examined longitudinally across the two waves (W1 and W2) of the COVID-19 pandemic, using standard cross-lagged models (Figure 5a). Models 5 and 6 included cross-lagged (paths a and b) and autoregressive effects (paths x, m, and y). No mediation effect of all three coping styles was found in the parallel cross-lagged Model 5 (Table 8). Although the emotion-oriented coping style during W2 was predictable based on perceived stress during W1, no coping style at W1 was found as a significant predictor of life satisfaction at W2. Weak stability over time (i.e., across two waves of the COVID-19 pandemic) was found for perceived stress, life satisfaction, task-oriented coping, and emotion-oriented coping style, while moderate stability was found for avoidance-oriented coping style in Model 5. The cross-lagged parallel mediation Model 5 demonstrates a perfect fit, as shown in Table 6.

When the reverse order was examined, with life satisfaction as a predictor of perceived stress (Model 6, Figure 5b), coping style during W1 was not significantly related to stress at W2, nor was life satisfaction at W1 related to coping style during W2 (see Table 8 for more details). Therefore, the mediating effect of coping style on the relationship between life satisfaction and perceived stress was not confirmed in the longitudinal approach. The goodness-of-fit measures were acceptable for Model 6 (Table 6).

**Figure 6:**
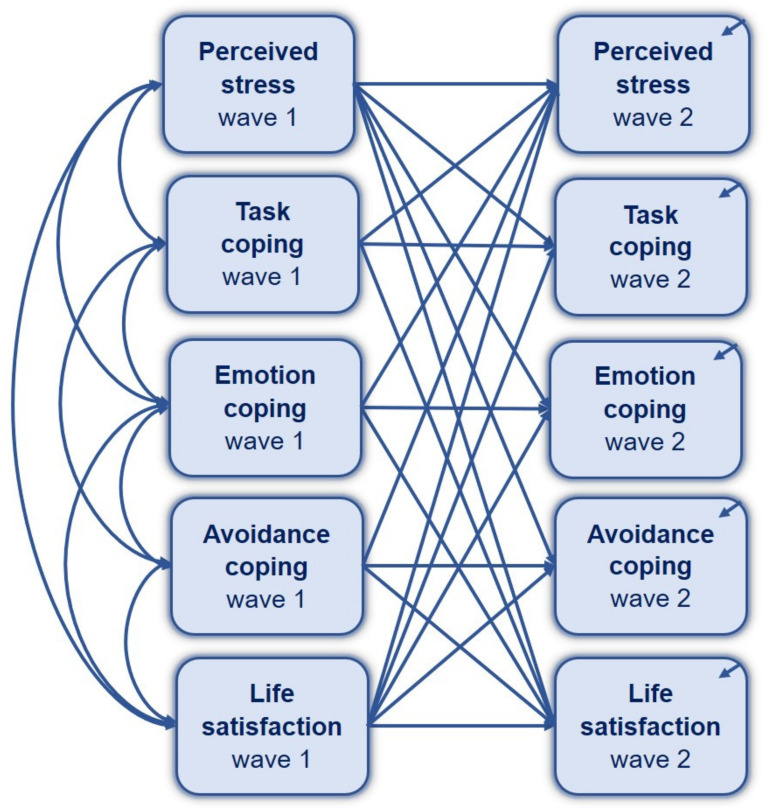
Conceptual reciprocal cross-lagged path model for a prospective study on the parallel mediation role of coping styles in the relationship between perceived stress and satisfaction with life.

The last analysis considered the reciprocal relationships between stress and life satisfaction, including the coping styles as mediators (Model 7, Figure 6). As shown in Table 8, significant regression coefficients were found only for the autoregressive path. Each variable during W2 in Model 7 could be predicted by the same variable during W1. Weak stability was presented by stress, life satisfaction, task-oriented, and emotion-oriented coping styles, whereas moderate stability was found for avoidance coping with stress. However, none of the variables during W2 could be predicted from any variable at W1. Model 7 demonstrates a perfect fit, as shown in Table 5.

## 4. Discussion

In this study, we examined for the first time the mediating effect of coping style on the relationship between perceived stress and life satisfaction in both cross-sectional and longitudinal models, in an extraordinary stressful situation such as the COVID-19 pandemic. Consistent with the transactional model of stress [53,73], we found significant changes between the first (W1) and second (W2) waves of the COVID-19 pandemic in perceived stress, life satisfaction, and some coping styles. The ANOVA showed that among the three coping styles, a larger change was reported for task-oriented coping. The frequency of using task-oriented and avoidance-oriented coping strategies significantly decreased, while the frequency of emotion-oriented coping slightly (but insignificantly) increased during W2 compared to W1. Furthermore, weak stability was noted in this study for stress, life satisfaction, and task- and emotion-oriented coping, whereas moderate stability was found for avoidance-oriented coping when the longitudinal cross-lagged model was examined. Both statistical methods (ANOVA and cross-lagged model) indicated that stress levels increased across pandemic waves, life satisfaction decreased, and the frequency of using selected coping strategies continuously changed over time.

The present results are consistent with previous research performed during the COVID-19 pandemic. A recent multi-cultural study showed that self-reported quality of life decreased over time, while perceived stress level and difficulty with emotion regulation increased significantly during the coronavirus pandemic [24]. Significant changes in stress and life satisfaction were found during the pandemic in various longitudinal studies [32,33,34,35,36]. Ruggieri et al. [32] performed a cross-lagged panel study during three waves (from one month before the lockdown), and they found increased levels of stress and decreased life satisfaction among Italian adults. A longitudinal study [34] on pre- and during-pandemic stressors and risk factors for distress changes showed that perceived stress during the pandemic was associated with pre-pandemic social stressors, stressful life events, low generalized trust, poor self-rated health, and some concurrent pandemic-related stressors and risk factors. Changes in lifestyle and economic disruptions, and loss of education or employment, were associated with greater increases in emotional distress. People who suffered high stress before the pandemic experienced increases in stress during the pandemic. During-pandemic stressors and hopelessness were found to be the strongest correlates of during-pandemic distress. Individuals distressed by the lockdown frequently used coping strategies such as seeking social support, engaging in distractions, and seeking professional help. Shanahan et al. [34] showed that several coping strategies significantly reduced distress during the pandemic, including keeping a daily routine, positive reappraisal/reframing, engaging in physical activity, acceptance, and keeping in contact with family and friends.

We found a 34% prevalence of high stress and 48% prevalence of low life satisfaction university students during W2 of the pandemic. In contrast, a systematic review found a 27% prevalence of high stress symptoms in the population during the COVID-19 pandemic [65]. However, a meta-analysis [66] indicated that very high stress was experienced by 45% of Chinese people during the crisis. Among Australian adults [67], 47% reported some degree of psychological distress during the first wave of the coronavirus pandemic, which was inversely related to coping strategies such as positive reframing, acceptance, and humor, and positively related to self-blame, venting, behavioral disengagement, and self-distraction. Research performed in Saudi Arabia found a 55.5% prevalence of high levels of stress during the quarantine, which was reduced by cognitive reappraisal and life satisfaction [68]. Numerous studies showed that the primary source of stress during the coronavirus pandemic was the significant lifestyle changes, related in particular to numerous restrictions and social isolation [17,38,39,40,41,42,43].

The partial mediating role of coping style in the relationship between stress and life satisfaction was confirmed in both cross-sectional studies during W1 (Model 1) and W2 (Model 2). A total of 70% of life satisfaction at W2 can be explained by predictor variables such as stress during W1 and W2, life satisfaction during W1, and by task-oriented and emotion-oriented coping styles during W2. However, among the three coping styles, emotion-oriented showed the strongest association with both stress and life satisfaction. In particular, a higher stress level was a predictor of the more frequent use of emotion-oriented coping strategies (positive association), which decreased life satisfaction (negative relation). This is consistent with previous studies [30,70,71], which indicated that university students reporting low life satisfaction, high stress, and most frequent use of emotion-oriented coping strategies during the pandemic. Marotta et al. [28] found an increase in negative and positive emotions, and a decrease in the quality of life among adult Italians during the first COVID-19 lockdown compared to reference data before the pandemic.

Higher stress levels were associated with a lower frequency of using task-oriented coping and a higher frequency of using avoidance-focused coping in the present study. This result is consistent with a previous study showing that people who experienced higher stress tended to use more passive rather than active coping strategies during pandemic [56,57]. When the environment is unpredictable and uncontrollable, task-oriented coping is perceived as less adaptive, whereas avoidance-focused coping strategies are more effective in reducing stress levels. This pattern was an adequate stress response in stressful life events or disasters [72].

The result of our mediation analysis is consistent to some extent with a previous study. Stapleton et al. [35] found that life satisfaction can be predicted by adaptive (positive association) and maladaptive (negative relation) coping, explaining 20% of the life satisfaction variance (*R*^2^ = 0.20) among teachers. Lardier et al. [74] showed that life satisfaction is negatively related to perceived stress and both suppressive (avoidance-oriented) and reactive (task-oriented) coping and is positively correlated with reflective (task-oriented) coping among Hispanic undergraduate students. Mediation analysis [74] indicated that perceived stress is indirectly related to life satisfaction through a reflective coping style. Higher levels of stress were associated with lower levels of reflective coping, which were related to lower life satisfaction. Both suppressive and reactive coping were not mediators in the relationship between perceived stress and life satisfaction. In contrast, we found a mediating role among all three coping styles: task-, emotion-, and avoidance-oriented. The differences between the results of the previous and current studies may be due to the various measurement tools used to assess coping with stress and cross-cultural variance.

Our findings also directly link stress to life satisfaction (Models 1 and 2) and conversely from life satisfaction to stress (Models 3 and 4). A stronger negative association was noted in the second wave of the pandemic than during the first wave. The relationship between stress and life satisfaction or other indices of well-being was previously reported in numerous studies [4,6,7,8,9,10,11,12,13,14]. Subjective well-being depends on many environmental and person-centered factors, including school and work, family and social life, and individual differences in affective states and personality traits and dispositions [1,2,3,4,5,91]. All these well-being-related factors may increase stress if changes in environment and lifestyle are perceived as a threat. Many studies indicated that stress and risk of mental disorders increased during the COVID-19 pandemic, whereas life satisfaction and quality of life decreased [15,16,17,18,19,20,21,22,23,24,25,26,27,28,29,30,31,32,33,34,35]. Studies indicated an inverse relationship between stress and various measures of well-being, e.g., [4,14,20,32,35], where higher levels of well-being corelated with lower levels of stress.

The reciprocal relationship of how life satisfaction affects stress through coping style was also tested cross-sectionally during W1 and W2. The results confirmed the partial mediation effect of task- and emotion-oriented coping styles. A high level of life satisfaction was related to an increased likelihood of using task-oriented coping, leading to decreased stress. Conversely, higher life satisfaction was a predictor of less frequent emotion-oriented coping, which could predict higher levels of perceived stress. Avoidance-oriented coping was not found to be a significant predictor of stress. Therefore, this coping style cannot play a mediating role in the relationship between life satisfaction and stress. This pattern was demonstrated at both measurement times, W1 and W2, during the COVID-19 pandemic.

The positive relationship of well-being with task-oriented coping style and negative relationship with emotion-oriented and avoidance-oriented was reported previously [35,58,59,60,61,62,63,64]. Boysan [58] demonstrated that life satisfaction is positively related to task-oriented (0.18, *p* < 0.01) and avoidance-oriented coping (0.08, *p* < 0.05), whereas negatively related to emotion-oriented coping (−0.20, *p* < 0.01). Xu et al. [64] found a relationship of high life satisfaction with high levels of positive coping (i.e., problem-solving, seeking social support, and positively rationalized explanation) and low levels of negative coping (enduring, escape, emotion venting, and wishful thinking/denial). Well-being was significantly and positively associated with approach coping and inversely related to avoidant coping behavior among UK adults during the first wave of the COVID-19 pandemic [60]. Engagement-related coping such as problem-solving was positively related to well-being, whereas disengagement coping such as blaming was negatively related. However, distraction and denial as coping strategies were not significantly associated with well-being among a large sample of the German population [61].

Stapleton et al. [35] found a moderate negative correlation of life satisfaction with perceived stress and a weak correlation with maladaptive coping, and a very weak but significant positive correlation between life satisfaction and adaptive coping among teachers. A regression analysis [35] showed that maladaptive (emotion-focused) coping is a strong positive predictor of psychological distress, whereas adaptive coping (task-oriented coping) is a weak negative predictor, explaining 45% of stress variance (*R*^2^ = 0.45). Problem-focused coping was considered an adaptive coping style since it was associated with better academic achievement among middle school students [92].

Another study showed that psychological well-being correlated negatively with avoidance strategy and positively with problem-solving coping among Italian university students [63]. Similarly, negative coping (i.e., high-stress perceptions, dysfunctional attitudes, and catastrophizing) showed a negative association with subjective well-being, whereas positive coping (hope, proactive coping style, and sense of humor) affected well-being positively [62]. Yan et al. [56] also showed that positive coping strategies are related to fewer symptoms of stress and mental health problems, whereas negative coping strategies aggravated emotional distress among a large sample of Chinese people during the first COVID-19 outbreak. Higher perceived adaptability to the COVID-19 pandemic was related to lower stress in a cross-sectional study in a sample of college students [93].

The result of this study is consistent to some extent with previous research on the mediating role of coping with stress [75]. Gori et al. [75] found a mediating effect of coping strategies on the relationship between life satisfaction and perceived stress in a cross-sectional study among Italian adults during the first wave of the COVID-19 pandemic. In particular, an indirect effect of life satisfaction on perceived stress was demonstrated via approach coping, positive attitude, and mature defenses, as indicated by a serial mediation model. Although Gori et al. [75] used a different tool to measure coping with stress (the Coping Orientations to Problems Experienced and the Forty-Item Defense Style Questionnaire), approach coping may be considered an alternative to task-oriented coping (in the CISS), positive attitude as an equivalent to inverse scores on the emotion-oriented coping scale, while mature defenses are similar (but only to some extend) to avoidance-oriented coping. Differences between previous [75] and the current study results in mediation analysis may be due to the various measurement methods used to assess coping styles and cross-cultural differences between the Italian adult population (with a mean age of 34 years old) and Polish university students (mean age of 23 years old).

Consistent with the hypothesis, longitudinal parallel mediation analysis across W1 and W2 during the pandemic was not confirmed. Although perceived stress during W1 was a significant positive predictor of emotion-oriented coping style during W2, no significant association was found between emotion-oriented coping style during W1 and life satisfaction during W2. Furthermore, neither task-oriented coping nor avoidance-oriented strategies were shown to be significantly related to stress or life satisfaction. When the reciprocal relationship between life satisfaction and stress via coping styles was examined, mediation was not found, and no coping style was associated with stress or life satisfaction. Notably, all autoregression pathways were significant in Models 5, 6 and 7. This means that all the variables at W2, included in Models 5 and 6, were predicted by the same variables at W1 (e.g., the perceived stress at W1 was a predictor of perceived stress at W2). Model 5, with perceived stress as a predictor of life satisfaction, received better fit indices than Model 6 with reciprocal relations. However, Model 7, with both paths from Model 5 and Model 6, showed a perfect and the best fit.

The results of this study indicate that a reciprocal association occurs between life satisfaction and stress, but it can only be observed at a given moment. We evidenced in cross-sectional studies during W1 and W2 that stress is a predictor of life satisfaction, and life satisfaction is a predictor of stress (Models 1 and 2, and Models 3 and 4, respectively). In addition, coping styles play a mediating role in the relationship between stress and life satisfaction, as well as in a reciprocal direction. In contrast, when the longitudinal design was examined, neither was perceived stress-related to life satisfaction (and vice versa), nor was coping style associated significantly with perceived stress or life satisfaction. As such, the mediation effect was not confirmed longitudinally. According to the transactional model of stress [53,73], stressful transactions between a person and their environment determines continuous changes in stress, coping, and well-being. Lifestyle changes and environmental stressful situations between W1 and W2 of the COVID-19 pandemic appear to be responsible for changes in the levels of the variables studied when comparing W1 to W2. This may explain why longitudinal mediation was not confirmed, although cross-sectional mediation was found twice during W1 and W2 of the pandemic. People adaptively change their coping strategies to the dynamic changes in stress levels and well-being as stressful events and lifestyle change during a pandemic. H5 was fully supported in this study.

### Limitations of the Study

There are some limitations that do not allow for generalization of our study’s results. First, the self-report measures of stress, life satisfaction, and coping styles may be biased to some extent. A future study can use experimental methods to assess the stress response. Although all measures were obtained during the COVID-19 pandemic, none include specific pandemic-related circumstances. Future studies may use more specific tools devoted to the stressful COVID-19 pandemic event. Although the sample size during the first and second cross-sectional studies was large, only 230 students participated in online surveys. The findings were collected at one technical university, so they may not represent all university students or the general population.

## 5. Conclusions

Perceived stress, life satisfaction, and coping styles significantly changed from the first to the second wave of the COVID-19 pandemic. University students considered changes in lifestyle and environment related to lockdown and adjusted their best coping strategies to these stressful events. As such, stress, and emotion-oriented and avoidance-oriented coping styles increased from W1 to W2, while life satisfaction and task-oriented coping decreased. These variables explained almost 70% of the variance in life satisfaction as stress and life satisfaction during W1, stress during W1 and W2, and emotion-oriented and task-oriented coping during W2. In the cross-sectional study, coping styles played a mediating role in the relationship between stress and life satisfaction at W1 and W2. However, in the longitudinal design, coping during W1 was not a useful predictor of life satisfaction or stress during W2. We found a reciprocal interrelation between stress and life satisfaction. Therefore, we found strong evidence aligned with the theory of the transactional model of stress. The findings provide new knowledge on our current understanding of stress and coping mechanisms concerning well-being. People try various coping strategies and choose the most effective at the moment and change them in response to the unstable environment. A vast repertoire of coping strategies, and flexibility in their selection, may be the best methods to effectively cope with stressful lockdowns during the COVID-19 pandemic and protect individuals against decreased well-being.

## Figures and Tables

**Table 1:** Demographic characteristics of the sample during the second wave of the COVID-19 pandemic.

Variable	Range	M	SD	n	%
Age	21–37	23.21	2.28	231	100
Gender					
Women				137	59.31
Men				92	39.83
Place of residence					
Village				107	46.32
Town				99	42.85
City				24	10.39
Agglomeration				1	0.43
Faculty of study					
Production Engineering and Logistics				45	19.48
Electrical Engineering, Automatics and Computer Science				65	28.14
Mechanical				40	17.32
Construction and Architecture				2	0.87
Economics and Management				7	3.03
Physical Education and Physiotherapy				72	31.17
Study level					
First level (Bachelor)				174	75.32
Second level (Master)				33	14.29
Five years’ master study				23	9.96
Doctoral				1	0.43
Type of study					
Full-time				200	86.58
Part-time				31	13.42
Study Year	1–5	2.78	1.02		
First				11	4.76
Second				102	44.16
Third				61	26.41
Fourth				41	17.75
Fifth				16	6.93

**Table 2:** Differences in life satisfaction, perceived stress, and coping styles between the first and second waves of the COVID-19 pandemic.

	Wave 1		Wave 2				
Variable	M	SD	M	SD	*F*(1, 230)	*p*	η*_p_*^2^
Life satisfaction	21.75	5.28	19.70	6.11	32.97	<0.001	0.13
Perceived stress	20.56	8.66	22.52	7.92	11.65	<0.001	0.05
Coping style							
Task-oriented	52.54	10.82	48.63	12.44	22.07	<0.001	0.09
Emotion-oriented	40.90	12.91	41.94	13.54	1.34	0.248	0.00
Avoidance-oriented	43.74	9.78	42.35	11.00	5.06	0.025	0.02

**Table 3:** Correlations matrix for all variables in W1 and W2 of the COVID-19 pandemic.

Variable		1	2	3	4	5	6	7	8	9
1	Life satisfaction W1									
2	Life satisfaction W2	0.56 ***								
3	Perceived stress W1	−0.38 ***	−0.21 **							
4	Perceived stress W2	−0.24 ***	−0.48 ***	0.45 ***						
Coping style										
5	Task-oriented W1	0.25 ***	0.23 ***	−0.27 ***	−0.16 *					
6	Task-oriented W2	0.12	0.23 ***	−0.21 **	−0.27 ***	0.42 ***				
7	Emotion-oriented W1	−0.37 ***	−0.22 ***	0.61 ***	0.31 ***	−0.05	−0.01			
8	Emotion-oriented W2	−0.27 ***	−0.42 ***	0.38 ***	0.64 ***	−0.07	0.06	0.47 ***		
9	Avoidance-oriented W1	0.13 *	0.15 *	0.20 **	0.10	0.16 *	0.09	0.38 ***	0.16 *	
10	Avoidance-oriented W1	0.05	−0.03	0.22 ***	0.24 ***	0.02	0.20 **	0.27 ***	0.46 ***	0.60 ***

Notes. W1 = wave 1 of the COVID-19 pandemic; W2 = wave 2 of the COVID-19 pandemic. * *p* < 0.05, ** *p* < 0.01, *** *p* < 0.001.

**Table 4:** Results of regression analysis for life satisfaction during the W2.

Variable	β	SE β	*b*	*SE b*	*t*(221)	*p*
Intercept			7.76	2.64	2.94	0.004
Life satisfaction W1	0.47	0.06	0.55	0.07	8.31	0.000
Perceived stress W1	0.20	0.07	0.14	0.05	2.99	0.003
Perceived stress W2	−0.32	0.07	−0.24	0.05	−4.49	0.000
Coping style						
Task-oriented W1	0.03	0.06	0.02	0.03	0.57	0.568
Task-oriented W2	0.12	0.06	0.06	0.03	2.01	0.046
Emotion-oriented W1	−0.03	0.07	−0.01	0.03	−0.38	0.705
Emotion-oriented W2	−0.16	0.08	−0.07	0.04	−2.01	0.046
Avoidance-oriented W1	0.12	0.07	0.08	0.04	1.80	0.072
Avoidance-oriented W1	−0.04	0.07	−0.02	0.04	−0.61	0.546

Notes. W1 = wave 1 of the COVID-19 pandemic; W2 = wave 2 of the COVID-19 pandemic.

**Table 5:** Parameter estimates for latent structural mediation models in cross-sectional approach.

Antecedent	Consequent	Path	Model 1 W1	Path	Model 2 W2
Stress	Life satisfaction	c’_1_	−0.185 *	c’_2_	−0.272 ***
Stress	Task coping	a_1_	−0.271 ***	a_4_	−0.274 ***
Stress	Emotion coping	a_2_	0.611 ***	a_5_	0.638 ***
Stress	Avoidance coping	a_3_	0.201 **	a_6_	0.239 ***
Task coping	Life satisfation	b_1_	0.136 *	b_4_	0.140 *
Emotion coping	Life satisfation	b_2_	−0.361 ***	b_5_	−0.324 ***
Avoidance coping	Life satisfation	b_3_	0.286 ***	b_6_	0.152 *

Notes. W1 = wave 1 of the COVID-19 pandemic; W2 = wave 2 of the COVID-19 pandemic. The values in the table are standardized regression coefficients (β). * *p* < 0.05, ** *p* < 0.01, *** *p* < 0.001.

**Table 6:** Fit indices for alternative models.

Model	ML Χ^2^	*df*	ML Χ^2^/*df*	*p*	SRMR	RMSEA	95% CI	CFI	TLI	NFI
1	6.091	1	6.091	0.014	0.027	0.149	0.054–0.271	0.979	0.787	0.976
2	11.541	1	11.541	0.001	0.035	0.214	0.116–0.332	0.965	0.645	0.962
3	6.091	1	6.091	0.014	0.027	0.149	0.054–0.271	0.979	0.787	0.976
4	11.541	1	11.541	0.001	0.035	0.214	0.116–0.332	0.965	0.645	0.962
5	10.183	13	0.783	0.679	0.022	0.000	0.000–0.052	1.000	1.012	0.988
6	21.914	13	1.686	0.057	0.032	0.055	0.000–0.093	0.989	0.963	0.975
7	3.653	6	0.609	0.723	0.014	0.000	0.000–0.063	1.000	1.021	0.993

Notes. ML = maximum likelihood; SRMR = standardized root mean square residual; root mean square error of approximation = RMSEA; CI–confidence interval; CFI = comparative fit index; normed fit index = NFI; the Tucker–Lewis index = TLI.

**Table 7:** Parameter estimates for latent structural mediation models in cross-sectional approach.

Antecedent	Consequent	Path	Model 3 W1	Path	Model 4 W2
Life satisfation	Stress	c’_1_	−0.141 *	c’_2_	−0.180 ***
Life satisfation	Task coping	a_1_	0.248 ***	a_4_	0.226 ***
Life satisfation	Emotion coping	a_2_	−0.373 ***	a_5_	−0.417 ***
Life satisfation	Avoidance coping	a_3_	0.135 *	a_6_	−0.031
Task coping	Stress	b_1_	−0.222 ***	b_4_	−0.274 ***
Emotion coping	Stress	b_2_	0.527 ***	b_5_	0.568 ***
Avoidance coping	Stress	b_3_	0.057	b_6_	0.025

Notes. W1 = wave 1 of the COVID-19 pandemic; W2 = wave 2 of the COVID-19 pandemic. The values in the table are standardized regression coefficients (β). * *p* < 0.05, *** *p* < 0.001.

**Table 8:** Parameter estimates for latent structural mediation models in the longitudinal approach.

Antecedent	Consequent	Model 5	Model 6	Model 7
Stress W1	Life satisfaction W2	0.018		0.054
Stress W1	Stress W2	0.450 ***	0.293 ***	0.357 ***
Stress W1	Task coping W2	−0.105		−0.107
Stress W1	Emotion coping W2	0.174 **		0.122
Stress W1	Avoidance coping W2	0.104		0.115
Task coping W1	Task coping W2	0.385 ***	0.410 ***	0.403 ***
Emotion coping W1	Emotion coping W2	0.337 ***	0.405 ***	0.363 ***
Avoidance coping W1	Avoidance coping W2	0.571 ***	0.591 ***	0.561 ***
Life satisfaction W1	Life satisfaction W2	0.479 ***	0.556 ***	0.499 ***
Task coping W1	Life satisfaction W2	0.075		−0.020
Emotion coping W1	Life satisfaction W2	−0.073		−0.086
Avoidance coping W1	Life satisfaction W2	0.101		0.022
Life satisfaction W1	Stress W2		−0.098	−0.057
Life satisfaction W1	Task coping W2		0.020	0.097
Life satisfaction W1	Emotion coping W2		−0.120	−0.110
Life satisfaction W1	Avoidance coping W2		−0.026	0.091
Task coping W1	Stress W2		−0.032	−0.056
Emotion coping W1	Stress W2		0.075	0.081
Avoidance coping W1	Stress W2		0.048	0.025

Notes. W1 = wave 1 of the COVID-19 pandemic; W2 = wave 2 of the COVID-19 pandemic. The values in the table are standardized regression coefficients (β). ** *p* < 0.01, *** *p* < 0.001.

## Data Availability

The datasets used and/or analysed during the current study are available from the corresponding author on reasonable request.
