# Correction: Rogowska et al. Changes in Stress, Coping Styles, and Life Satisfaction between the First and Second Waves of the COVID-19 Pandemic: A Longitudinal Cross-Lagged Study in a Sample of University Students. *J. Clin. Med.* 2021, *10*, 4025

**DOI:** 10.3390/jcm11010276

**Published:** 2022-01-05

**Authors:** Aleksandra Maria Rogowska, Cezary Kuśnierz, Dominika Ochnik

**Affiliations:** 1 Institute of Psychology, Faculty of Social Sciences, University of Opole, 45-052 Opole, Poland; 2 Faculty of Physical Education and Physiotherapy, Opole University of Technology, 45-758 Opole, Poland; c.kusnierz@po.edu.pl; 3 Faculty of Medicine, University of Technology, 40-555 Katowice, Poland; dominika.ochnik@wst.pl

The authors wish to make the following corrections to this paper [1]:

## 1. Change in Abstract

The sentence “The task-oriented and avoidance-oriented coping styles can play a mediating role in the reciprocal relationship between life satisfaction and perceived stress during W1 and W2 of the pandemic” is changed into “The task-oriented and emotion-oriented coping styles can play a mediating role in the reciprocal relationship between life satisfaction and perceived stress during W1 and W2 of the pandemic”.

## 2. Change in Main Body Paragraph

In the Discussion section (8th paragraph), the following sentence “Avoidance-oriented coping was not found to be a significant predictor of stress. Therefore, this coping style plays a mediating role in the relationship between life satisfaction and stress.” is modified into “Avoidance-oriented coping was not found to be a significant predictor of stress. Therefore, this coping style cannot play a mediating role in the relationship between life satisfaction and stress”.

The authors apologize for any inconvenience caused and state that the scientific conclusions are unaffected. The original article has been updated.
